# 2021–2022 state of our JCMR

**DOI:** 10.1186/s12968-022-00909-6

**Published:** 2022-12-31

**Authors:** Warren J. Manning

**Affiliations:** Departments of Medicine (Cardiovascular Division) and Radiology, Beth Israel Deaconess Medical Center, Harvard Medical School and JCMR Editorial Office, Boston, MA 02215 USA

## Abstract

In 2021, there were 136 articles published in the *Journal of Cardiovascular Magnetic Resonance* (*JCMR*), including 122 original research papers, six reviews, four technical notes, one Society for Cardiovascular Magnetic Resonance (SCMR) guideline, one SCMR position paper, one study protocol, and one obituary (Nathaniel Reichek). The volume was up 53% from 2020 (n = 89) with a corresponding 21% decrease in manuscript submissions from 435 to 345. This led to an increase in the acceptance rate from 24 to 32%. The quality of the submissions continues to be high. The 2021 JCMR Impact Factor (which is released in June 2022) markedly increased from 5.41 to 6.90 placing us in the top quartile of Society and cardiac imaging journals. Our 5 year impact factor similarly increased from 6.52 to 7.25. Fifteen years ago, the *JCMR* was at the forefront of medical and medical society journal migration to the Open-Access format. The Open-Access system has dramatically increased the availability and *JCMR* citation. Full-text article requests in 2021 approached 1.5 M!. As I have mentioned, it takes a village to run a journal. *JCMR* is very fortunate to have a group of very dedicated Associate Editors, Guest Editors, Journal Club Editors, and Reviewers. I thank each of them for their efforts to ensure that the review process occurs in a timely and responsible manner. These efforts have allowed the *JCMR* to continue as the premier journal of our field. My role, and the entire editorial process would not be possible without the ongoing high dedication and efforts of our managing editor, Jennifer Rodriguez. Her premier organizational skills have allowed for streamlining of the review process and marked improvement in our time-to-decision (see later). As I conclude my 6th and final year as your editor-in-chief, I thank you for entrusting me with the *JCMR* editorship and appreciate the time I have had at the helm. I am very confident that our *Journal* will reach new heights under the stewardship of Dr. Tim Leiner, currently at the Mayo Clinic with a seamless transition occurring as I write this in late November*.* I hope that you will continue to send your very best, high quality CMR manuscripts to *JCMR,* and that our readers will continue to look to *JCMR* for the very best/state-of-the-art CMR publications.

## Background

The *JCMR* is the official publication of the Society for Cardiovascular Magnetic Resonance (SCMR). In 2021, the *JCMR* published 136 articles published in the *Journal of Cardiovascular Magnetic Resonance* (*JCMR*), including 122 original research papers, six reviews, four technical notes, one Society for Cardiovascular Magnetic Resonance (SCMR) guideline, one SCMR position paper, one study protocol, and one obituary (Nathaniel Reichek). The 2021 publication volume was up 53% from 2020 (n = 89) with a corresponding 21% decrease in manuscript submissions from 435 to 345. This led to an increase in the acceptance rate from 24 to 32% (the slight mathematical difference in acceptance/submissions is related to submission year and publication year). As might be expected, COVID-19 publications [1–8] and COVID-19 vaccination publications (8) were plentiful, with 8 published in 2021.

In July 2018, the article processing charge (APC) structure changed with SCMR members who are the submitting author paying an APC of only $500, presenting an 82% discount to the full $2680 APC. Reduced APC fees are also available to those from BMC membership institutions, submitting authors from lower income countries, and for those who request a waiver due to financial hardship. APCs are waived for invited reviews and for Society publications.

**Fig. 1:**
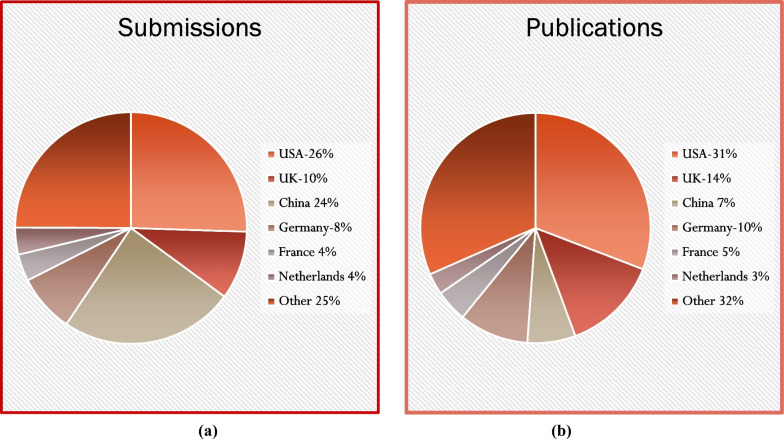
Pie charts for 2021 *Journal of Cardiovascular Magnetic Resonance (JCMR*) origin country for (**A**) submissions and (**B**) publications

As for 2020, in 2021, the United States (26%) and China (24%) were the source of 50% of all *JCMR* publications followed by the United Kingdom (10% and Germany (8%). The top three countries for publications were the United States (31%), United Kingdom (14%) and Germany (10%) (Fig. 1).

### Impact factor

Though only one of many journal metrics and not a consideration in our review process, the Impact Factor calculated by Clarivate Analytics is nonetheless a well-recognized metric with which many readers are familiar and is a metric often considered by both authors and readers for submitting and reading manuscripts. I am pleased to report that the 2021 *JCMR* Impact Factor (which was released in June 2022 and is based on manuscripts published in 2019 (n = 430) and 2020 (n = 633) that were cited in 2021) increased from 5.41 to 6.90!. This impact factor means that the *JCMR* papers published in 2019 and 2020 were cited on average 6.90 times in 2021. This puts *JCMR* well positioned in the top quartile (34/142–previously 37/142) of journals in the broad categories of “Cardiac and Cardiovascular Systems” and the top quintile (21/133–previously 20/133) of “Radiology, Nuclear Medicine and Medical Imaging.” Our 2021 5 year impact factor similarly increased from 6.52 to 7.25. The 2022 *JCMR* impact factor will be released in June 2023.

Perhaps more important than the Impact Factor is the frequency that *JCMR* articles are accessed. Our open-access format allows for much greater visibility for our authors with the 2021 *JCMR* annual digital downloads now approaching 1,500,000!!—a threshold not achievable with a subscription/print publication of a relatively small Society journal. Open-access has “leveled the playing field” so that an electronic search allows *JCMR* manuscripts to rise to awareness and to then be downloaded without cost. This is a great benefit to our readers, to the greater scientific community, and to our authors. Data analytics provided by our publisher, BMC, indicate that the vast majority (72%) of on line manuscript searches are identified from a Google, 9% directly from the *JCMR* web site, 4% from Pubmed. The largest number of searches are from Europe (38%) followed by the United States (28%).

## *JCMR* editor-in-chief leadership

**Fig. 2:**
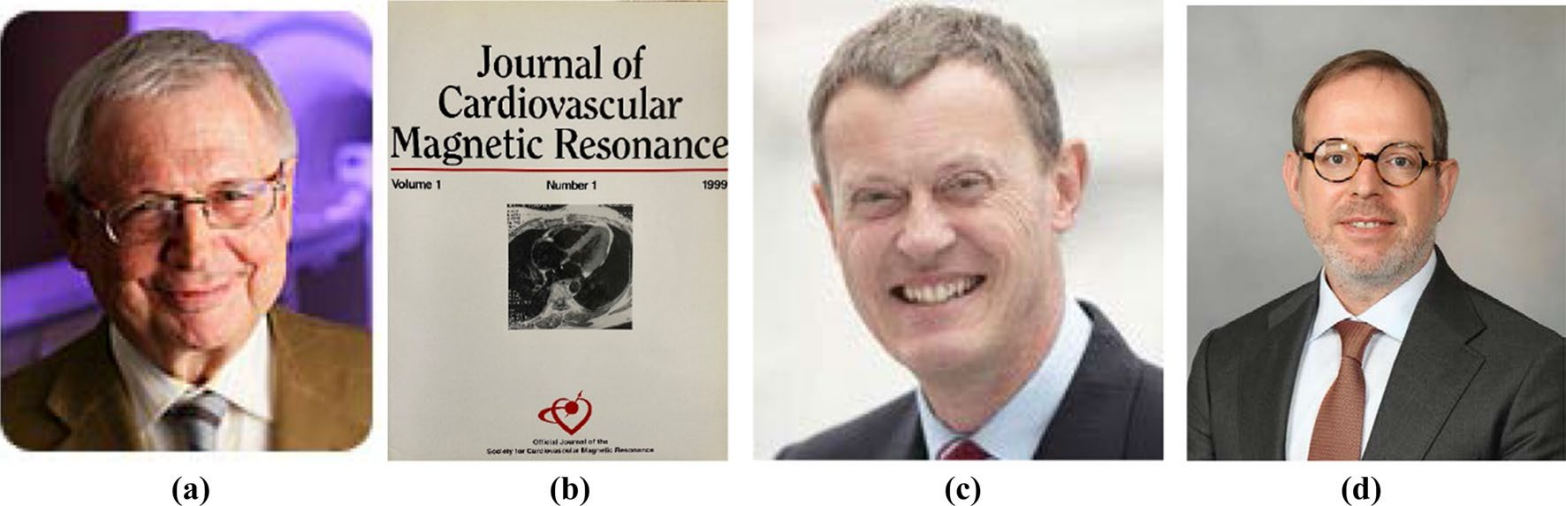
**A** Inaugural (1999–2005) *JCMR* editor-in-chief, Dr. Gerald M. Pohost. **B** first *JCMR print* issue (**C**) second editor-in-Chief, Professor Dudley Pennell (2006–2016), **D** Dr. Tim Leiner will be the 4th JCMR editor-in-chief effective 1/1/2013

Dr. Gerald Pohost (Fig. 2) from the University of Alabama at Birmingham and University of Southern California, Los Angeles, CA, USA was the *JCMR* inaugural editor-in-chief (1999). During his tenure, the *JCMR* was published in print format by Marcel Dekker, Inc (Fig. 2). In 2007, he was succeeded by Professor Dudley Pennell (Fig. 2) of the Royal Brompton Hospital, London, England. Since December 2016, the *JCMR* editorial office has been located at the Beth Israel Deaconess Medical Center, Boston, MA, USA under my leadership. My 6 year term will end at the end on December 31, 2022. We are well underway for an organized transition to the 4th JCMR Editor-in-Chief, Dr. Tim Leiner, currently at the Mayo Clinic, Rochester, Minnesota, USA. Throughout this transition, you can continue to contact the *JCMR* editor-in-chief by using the same email: jcmreditor@scmr.org.

## 2021 *JCMR* editorial and management team

The *JCMR* Associate Editors (Table 1) reflect the international and diverse spectrum of the CMR and SCMR field. Dr. Long Ngo (USA) continues to serve as our statistical editor. Drs. Juan Lopez-Mattei (USA) and Purvi Parwani (USA) are busy every week disseminating *JCMR* news as our Social Media/Twitter editors. Tim has elected to keep most of the current team in place and will be adding several Associate Editors. Stay tuned!

**Table 1:** JCMR associate editors, statistical editor, journal club editors, and social media editors

**Associate editors**	
Rene Botnar	Pontificia Universidad Católica, Chile/King’s College London, UK
John Greenwood	University of Leeds, UK
Yuchi Han	Ohio State University, USA
Dara Kraichman	Johns Hopkins University School of Medicine, USA
Robert Lederman	National Institutes of Heart, Lung, and Blood Institute, USA
Tim Leiner	Mayo Clinic, USA
Reza Nezafat	Beth Israel Deaconess Medical Center, USA
Amit Patel	University of Virginia, USA
Joshua Robinson	Northwestern University, USA
Connie Tsao	Beth Israel Deaconess Medical Center, USA
**Statistical editor**	
Long Ngo	Beth Israel Deaconess Medical Center, USA
**Journal Club Editors**	
Scott Flamm	Cleveland Clinic, USA
Raymond Kwong	Brigham and Women’s Hospital, USA
Matthias Stuber	University of Lausanne, Switzerland
**Social Media Editors**	
Juan Lopez-Mattei	Lee Health, USA
Purvi Parwani	Loma Linda University Health, USA

**Fig. 3:**
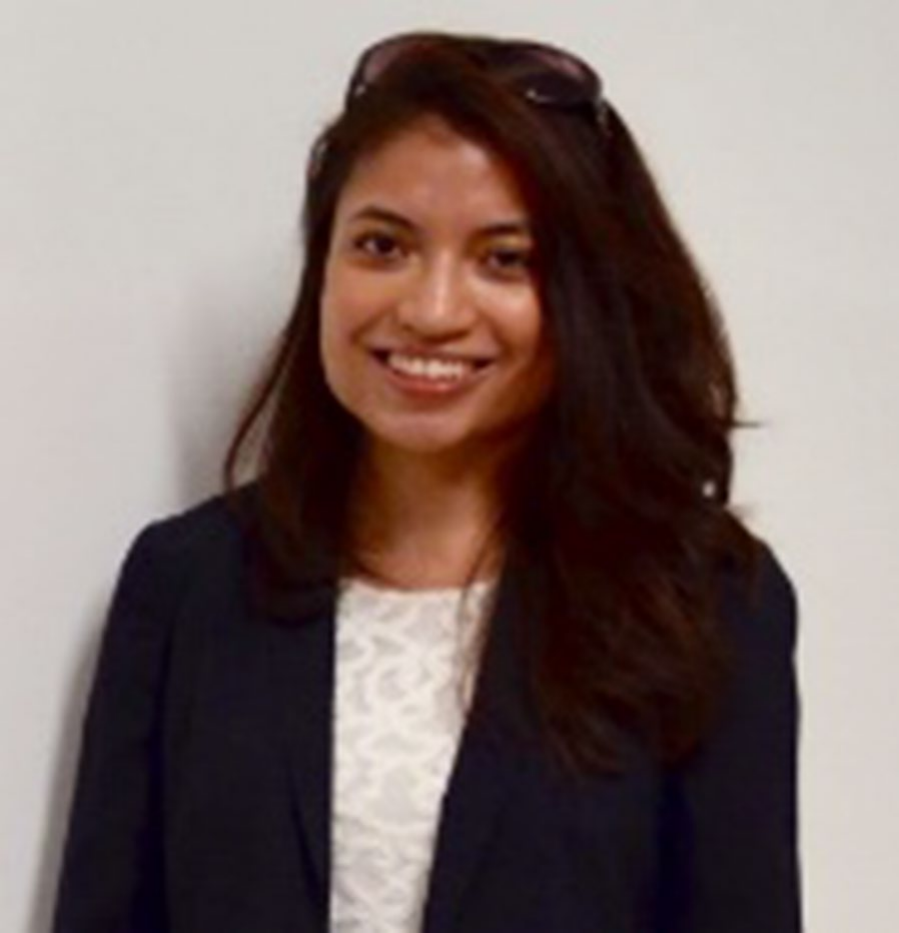
Jennifer Rodriguez*, JCMR* managing editor since January 2021

Jennifer Rodriguez (jcmroffice@scmr.org) has been our managing editor since January 2021 (Fig. 3). Jennifer has made tremendous progress in keeping me and the entire manuscript review process organized and on schedule. As a result, we have seen a marked decrease in our time to first decision time from a mean of 60 days in 2019 and 2020 to ≤ 40 days since she took the managing editor position in January 2021. I hope our authors have felt this tangible difference. We are fortunate that Jennifer has agreed to continue in her *JCMR* managing editorial role with Dr. Leiner.

## 2021 *JCMR* Journal Club—now with CME!

**Fig. 4:**
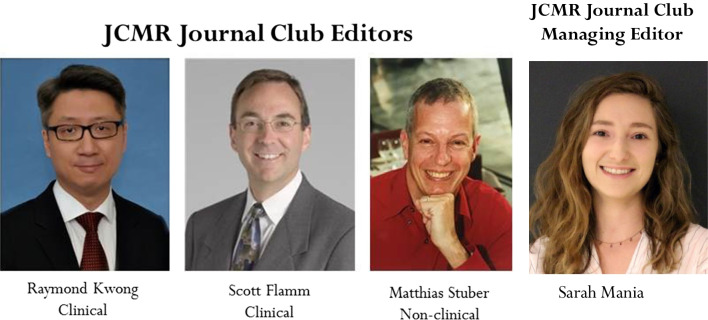
2020–2022 JCMR Journal Club editors: Drs. Raymond Kwong, Scott Flamm, Matthias Stuber. Ms. Sarah Mania has been the JCMR Journal Club Managing Editor since mid-2021

A highlight of 2021 was the second season of our *JCMR* Journal. These monthly one-hour webinars are held on the 2nd Wednesday of the month at 11am ET. A link for the monthly registration is on the JCMR (https://jcmr-online.biomedcentral.com/) and SCMR (www.scmr.org) websites. For three years, these monthly *JCMR* Journal Clubs have been moderated by one of our three Journal Club Editors, Drs. Scott Flamm (clinical), Raymond Kwong (clinical) and Matthias Stuber (non-clinical) (Fig. 4). On a rotating basis, each editor choses a manuscript that was recently published in *JCMR.* After a brief Journal Club Editor introduction of the topic, the presenting author has a 25–30 min presentation followed by a spirited 30 min discussion. We continue to offer continuing medical education (CME) for reading the manuscript and for July–December 2022 started providing CME for Journal Club *attendance.* CME for our *JCMR* Journal Club is another free benefit for SMCR members. Please join your colleagues every month for an informative presentation and discussion. Don’t worry if you missed one. Recordings of the monthly webinars and a CME journal link are provided on the *JCMR* web site. Check them out! While you can receive CME for reading the manuscript at any time, you can only receive CME for journal club *attendance* when participating in the live event.

Like other JCMR activities, the *JCMR* Journal Club is a village effort. In addition to our 3 talented Journal Club editors, I very much appreciate the strong administrative assistance of Sarah Mania (Fig. 4) for the past 18 months. Sarah was responsible for coordinating registration, the speaker presentations, CME, Zoom operations and recording, and subsequent posting of the monthly *JCMR* Journal Club recording on the SCMR website. The 2021 JCMR Journal Club selections were on a wide variety of topics (Table 2).

**Table 2:** 2020 Monthly JCMR Journal Club Editor, Presenter, Manuscript. Continuing medical education (CME) is offered for reading of the manuscript and is a complimentary benefit for SCMR members

Date	Journal Club Editor	Presenter	Manuscript
1/13/2021	Raymond Kwong	Tomaz Podlesnikar	Left ventricular functional recovery of infarcted and remote myocardium after ST-segment elevation myocardial infarction (METOCARD-CNIC randomized clinical trial substudy [22]
2/10/2021	Matthias Stuber	Lenhard Pennig	Clinical application of free-breathing 3D whole heart late gadolinium enhancement cardiovascular magnetic resonance with high isotropic spatial resolution using Compressed SENSE [23]
3/10/2021	Scott Flamm	Claire Raphael (Sanjay Prasad)	Cardiovascular magnetic resonance predictors of heart failure in hypertrophic cardiomyopathy: the role of myocardial replacement fibrosis and the microcirculation [24]
4/14/2021	Raymond Kwong	Theo Pezel (Jerome Garot)	Long-term prognostic value of stress perfusion cardiovascular magnetic resonance in patients without known coronary artery disease [25]
5/12/2021	Matthias Stuber	Thu-Thao Le	Multiparametric exercise stress cardiovascular magnetic resonance in the diagnosis of coronary artery diseases: the EMPIRE trial [26]
6/9/2021	Scott Flamm	Ying Zhang (Yuchi Han)	Comparing cardiovascular magnetic resonance strain software packages by their abilities to discriminate outcomes in patients with heart failure with preserved ejection fraction [27]
7/14/2021	Raymond Kwong	Alessia Pepe	Myocardial iron overload by cardiovascular magnetic resonance native segmental T1 mapping: a sensitive approach that correlates with cardiac complications [28]
8/10/2021	Matthias Stuber	Sorin Giusca (Greg Korosoglou)	Multi-parametric assessment of left ventricular hypertrophy using late gadolinium enhancement, T1 mapping and strain-encoded cardiovascular magnetic resonance [29]
9/8/2021	Scott Flamm	Robert Holtackers	Dark-blood late gadolinium enhancement cardiovascular magnetic resonance for improved detection of subendocardial scar: a review of current techniques [11]
10/13/2021	Raymond Kwong	Hakan Arheden	Pulmonary blood volume measured by cardiovascular magnetic resonance: influence of pulmonary transit time methods and left atrial volume [30]
11/10/2021	Matthias Stuber	Robert Edelman	Dark blood cardiovascular magnetic resonance of the heart, great vessels, and lungs using electrocardiographic-gated three-dimensional unbalanced steady-state free precession [31]
12/8/2021	Scott Flamm	Shingo Kato	Cardiovascular magnetic resonance assessment of coronary flow reserve improves risk stratification in heart failure with preserved ejection fraction [32]

## Manuscript review process, omissions, and suggestions

I reviewed the manuscript submission process in my report earlier this year [9] and will not repeat that outline.

All manuscripts are submitted and processed through the http://www.jcmr-online.org website. I encourage all authors to closely follow the guidelines so as not to delay the review process. By far, the most error that leads to review delay continues to be the omission of the names and contact information for *at least two suggested reviewers* in their submission documents. I ask authors to use *JCMR* preferred abbreviations (Table 3; https://jcmr-online.biomedcentral.com/submission-guidelines/preparing-your-manuscript/abbreviations) and to use the terms “CMR” and “cardiovascular magnetic resonance” rather than “cardiac magnetic resonance” or “cardiac MRI.” While the abbreviation issue does not delay the review, it adds additional burden to the prepublication editing process.

**Table 3:** JCMR preferred abbreviations

3D	Three-dimensional
4Ch	Four chamber
4D	Four-dimensional
4DF	Four-dimensional flow
6MWT	Six minute walk test
A	Area
A2C	Apical two chamber
A4C	Apical four chamber
*AA*	*Aortic arch*
*AA*	*Adductor artery*
AAA	Abdominal aortic aneurysm
AAo	Ascending aorta
AAOCA	Anomalous aortic origin of the coronary arteries
AAP	American academy of pediatrics
AAR	Area at risk
ABI	Ankle-brachial index
AC	Arrhythmic cardiomyopathy
ACA	Anterior cerebral artery
ACAOS	Anomalous coronary artery origin from the opposite sinus
ACAR	Acute cardiac allograft rejection
ACC	American College of Cardiology
ACCF	American College of Cardiology Foundation
ACDC	Automated Cardiac Diagnosis Challenge
ACEI	Angiotensin converting enzyme inhibitor
aCNR	Apparent contrast-to-noise ratio
ACS	Acute coronary syndrome
*ACR*	*American College of Radiology*
*ACR*	*Acute cardiac rejection*
ACS	Acute coronary syndrome
*AD*	*Aortic distensibility*
*AD*	*Aortic dissection*
ADAM	Adaptive moment estimation algorithm
ADC	Apparent diffusion coefficientddddddddddd
ADMM	Alternating direction method of multipliers
ADP	Adenosine diphosphate
ADT	Appropriate device therapy
AE	Adverse event
*AF*	*Atrial fibrillation*
*AF*	*Atlas Forests*
AFD	Anderson-Fabry disease
AFP	Adiabatic full passage
AG	Attention gates
AHA	American Heart Association
AHP	Adiabatic half passage
AI	Artificial intelligence
AIC	Akaike’s information criteria
AIF	Arterial input function
AIM	Annular inflow method
AKI	Acute kidney injury
AL	Amyloid light chain
ALM	Appendicular lean mass
ALSA	Aberrant left subclavian artery
aLV	Apical left ventricle
AM	Acute myocarditis
AMA	American Medical Association
AMI	Acute myocardial infarction
AML	Anterior mitral leaflet
AMR	Antibody mediated rejection
ANCA	Anti-neutrophil cytoplasmic antibody
ANCOVA	Analysis of covariance
ANOVA	Analysis of variance
AOA	Anatomic orifice area
AoR	Aortic root
*AP*	*Anterior–posterior*
*AP*	*Aorto-pulmonary*
ApA	Apical angle
APC	Aortopulmonary collateral
APEF	Apical ejection fraction
APMHR	Age predicted maximal heart rate
APVD	Anomalous pulmonary venous drainage
ARB	Angiotensin receptor blocker
ARD	Autoimmune rheumatic diseases
ARDS	Acute respiratory distress syndrome
ARoot	Aortic root
ART	Antiretroviral therapy
ARVC	Arrhythmogenic right ventricular cardiomyopathy
AS	Aortic stenosis
aSNR	Apparent signal-to-noise ratio
*ASD*	*Atrial septal defect*
*ASD*	*Average surface distance*
ASE	American Society of Echocardiography
ASI	Aortic size index
ASL	Arterial spin labeling
ASNC	American Society of Nuclear Cardiology
ASO	Arterial switch operation
AT2R	Angiotensin 2 receptor
AT1R	Angiotensin 1 receptor
*ATP*	*Adenosine triphosphate*
*ATP*	*Antitachycardia pacing*
ATTR	Amyloid transthyrein (amyloidosis)
*AUC*	*Appropriate use criteria*
*AUC*	*Area under the curve*
AVA	Aortic valve area
AVAI	Aortic valve area index
*AVC*	*Arrhythmic ventricular cardiomyopathy*
*AVC*	*Aortic valve closure*
AVI	Aorto-vertebral interface
AVM	Arteriovenous malformation
AVO_2_	Arteriovenous oxygen
AVPD	Atrioventricular plane descent
AVR	Aortic valve replacement
AVVR	Atrioventricular valve regurgitation
BA	Basilar artery
BAV	Bicuspid aortic valve
*BB*	*Black blood*
*BB*	*Bright blood*
BBTI	Black blood thrombus imaging
BCA	Brachiocephalic artery
BCS	Blind compressed sensing
BCW	Backwards compression wave
BDG	Bidirectional Glenn
BH	Breath hold
BIC	Bayesian information criteria
BiPAP	Bi-level positive airway pressure
BiV	Biventricular
bLV	Basal left ventricle
BMC	Blood-to-myocardial contrast
BMD	Becker muscular dystrophy
BMI	Body mass index
BMV	Bioprosthetic mitral valve
BNP	Brain natriuretic peptide
BOLD	Blood-oxygen dependent contrast
BOOST	Bright-blood and black-blOOd phase SensiTive inversion recovery
BP	Blood pressure
BPD	Bronchopulmonary dysplasia
BPM	Beats per minute
BSA	Body surface area
bSSFP	Balanced steady state free precession
BUN	Blood urea nitrogen
Bvf	Fractional tissue blood volume per cardiac tissue volume
*BW*	*Band width*
*BW*	*Body weight*
C	Compacted
C-SENSE	Compressed sensitivity encoding
CA	Cardiac amyloidosis
*CAA*	*Coronary artery anomaly*
*CAA*	*Coronary artery aneurysm*
CABG	Coronary artery bypass graft
CAC	Coronary artery calcification
CAD	Coronary artery disease
CAP	Cardiac Atlas Project
CATCH	Coronary atherosclerosis T1w characterization with integrated anatomic reference
CAV	Coronary allograft vasculopathy
CAVI	Cardio-ankle vascular index
CBC	Complete blood count
CBCMR	Certification Board of Cardiovascular Magnetic Resonance
CBF	Coronary blood flow
CCA	Common carotid artery
CCMRA	Coronary cardiovascular magnetic resonance angiography
ccTGA	Congenitally corrected transposition of the great arteries
CDC	United States Centers for Disease Control and Prevention
cDTI	Cardiac diffusion tensor imaging
*CE*	*Contrast enhanced*
*CE*	*Cholesterol esthers*
*CE*	*Cardiac events*
*CE*	*Conformité* *Européenne*
*CE*	*Continuing education*
CE-MARC	Clinical Evaluation of Magnetic Resonance Imaging in Coronary Heart Disease
*CEA*	*Carotid endarterectomy*
*CEA*	*Cost effectiveness analysis*
CEF	Coronary endothelial function
CEP	Clinical end-point
CETP	Cholesterylester transfer protein
cf-PWV	Carotid-femoral pulse wave velocity
CF	Center frequency
CFA	Common femoral artery
cFA	Compartment fractional anisotropy
CFD	Computational flow dynamics
CFR	Coronary flow reserve
CHD	Congenital heart disease
CHESS	Chemical shift selective saturation
CHIP	Coronary hyper-intense plaque
*CI*	*Confidence interval*
*CI*	*Cardiac index*
CIA	Common iliac artery
CIC	Cardiac iron concentration
CIED	Cardiac implanted electronic device
CIF	Cumulative incidence function
CircE	Circulatory efficiency
CK	Creatine kinase
CKD	Chronic kidney disease
CLIO	Cross-linked iron oxide
cMD	Compartment mean diffusivity
CMD	Coronary microvascular dysfunction
CME	Continuing medical education
C_MET_	Cardiac metastases
*CMP*	*Central mean pressure*
*CMP*	*Cardiomyopathy*
CMR	Cardiovascular magnetic resonance
*CMRA*	*Coronary magnetic resonance angiography*
*CMRA*	*Cardiovascular magnetic resonance angiography*
CMRS	Cardiovascular magnetic resonance spectroscopy
CMRV	Cardiovascular magnetic resonance venography
CMS	Centers for Medicare and Medicaid Services
CMV	Cytomegalovirus
CNN	Convolutional neural networks
CNR	Contrast-to-noise ratio
CNS	Central nervous system
CO	Cardiac output
CO_eff_	Effective cardiac output
CoA	Coarctation of aorta
COCATS	Core Cardiovascular Training Statement
CoG	Center of gravity
CoM	Center of mass
COPD	Chronic obstructive pulmonary disease
COR	Class of recommendation
CorCTA	Coronary computed tomography angiography
COSMOS	Calculation of susceptibility through a multiple-oreintation sampling
CoV	Coefficient of variation
COVID-19	Coronavirus disease 2019
CP	Circulatory power
CPET	Cardiopulmonary exercise testing
CPK	Creatine phosphokinase
CPO	Cardiac power output
CPR	Curved planar reconstruction
Cr	Creatinine
CR	Contrast ratio
CRF	Cardiorespiratory fitness
CRP	C-reactive protein
CRT	Cardiac resynchronization therapy
*CS*	*Compressed sensing*
*CS*	*Coronary sinus*
*CS*	*Circumferential strain*
CSA	Cross-sectional area
CSBF	Coronary sinus blood flow
CSDE	Chemical shift displacement error
CSF	Cerebral spinal fluid
CSI	Chemical shift imaging
CSPAMM	Complementary spatial modulation of magnetization
CT	Computed tomography
CT-FT	Computed tomography feature tracking
cT1	Corrected T1
CTA	Computed tomography angiography
CTD	Connective tissue disease
CTEPH	Chronic thromboembolic pulmonary hypertension
cTn	Cardiac troponin
CTO	Chronic total occlusion
CTRCD	Cancer therapy-related cardiac dysfunction
CuCo	Cusp to commissure
CuCu	Cusp to cusp
CV	Cardiovascular
CVA	Cerebrovascular attack
cVAE	Conditional variational autoencoder
CVD	Cardiovascular disease
CVF	Collagen volume fraction
CVO	Combined ventricular output
CVP	Central venous pressure
CVR	Cerebrovascular resistance
D	Distance
D-TGA	Dextro-transposition of the great arteries
*dAA*	*Distal aortic arch*
*dAA*	*Distal ascending aorta*
DA	Descending aorta
DAA	Double aortic arch
DB	Dark blood
DBP	Diastolic blood pressure
*DC*	*Distensibility coefficient*
*DC*	*Diagnostic confidence*
DCE	Dynamic contrast enhancement
DCI	Diffusion compartment imaging
DCM	Dilated cardiomyopathy
DCMR	Dobutamine stress cardiovascular magnetic resonance
DCMRL	Dynamic contrast cardiovascular magnetic resonance lymphangiography
DCS	Diastolic circumferential strain
DD	Ductus diverticulum
dDA	Distal descending aorta
dDNP	Dissolution dynamic nuclear polarization
De	Dean number
DENSE	Displacement encoding with stimulated echoes
DESPOT	Driven equilibrium single pulse observation of T1
DEXA	Dual-energy x-ray absorptiometry
DICOM	Digital imaging and communications in medicine
DIF	Diffuse interstitial fibrosis
DIR	Double inversion recovery
DIRV	Double inlet right ventricle
DL	Deep learning
DLCO	Diffusion lung capacity for carbon monoxide
DLV	Dominant left ventricle
DM	Diabetes mellitus
DMD	Duchenne’s muscular dystrophy
DMF	Diffuse myocardial fibrosis
dNAV	Diaphragmatic navigator
DORV	Double outlet right ventricle
DRA	Dark rim artifact
DRV	Dominant right ventricle
*DSA*	*Digital subtraction angiography*
*DSA*	*Donor specific antibodies*
DSC	Dice similarity coefficient
DSE	Dobutamine stress echocardiography
DORV	Double outlet right ventricle
dp-SIR	Dentate nucleus to pons signal intensity ratio
DRA	Dark rim artifact
DRAPR	Deep learning radial acceleration with parallel reconstruction
DSA	Digital subtraction angiography
DSC	DICE similarity coefficient
DSS	Dahl salt-sensitive
DSVR	Deformable slice to volume registration
DTA	Descending thoracic aorta
DTI	Diffusion tensor imaging
DUS	Doppler ultrasound
DTPA	Diethylenetriaminepentaacetic acid
DTW	Dynamic time warp
DVQ	Diastolic vorticity quotient
DVD	Double vessel disease
DVT	Deep venous thrombosis
*DW*	*Diffusion weighted*
*DW*	*Dry weight*
DWI	Diffusion weighted imaging
E2A	Secondary eigenvector
e’	Early diastolic velocity
*Ea*	*Effective elastance*
*Ea*	*Arterial elastance*
EACVI	European Association of Cardiovascular Imaging
EAM	Electroanatomic map
EBV	Epstein-Barr virus
ECA	External carotid artery
Ecc	Circumferential strain
ECC	Extracardiac conduit
ECF	Extracellular fluid
ECG	Electrocardiogram
ECM	Extracellular matrix
ECMO	Extracorpeal membrane oxygenation
ECV	Extracellular volume fraction
ECVm	Measured extracellular volume fraction
ECVsyn	Synthetic extracellular volume fraction
ED	End-diastole
EDD	End-diastolic dimension
EDS	Ehlers-Danlos syndrome
EDV	End-diastolic volume
EDVI	End-diastolic volume index
EED	Endocardial edge delineation
EEM	External elastic membrane
Ees	End-systolic elastance
*EF*	*Ejection fraction*
*EF*	*Emptying fraction*
EF1	First phase ejection fraction
eCNR	Estimated contrast-to-noise ratio
EGE	Early gadolinium enhancement
EGEr	Early gadolinium enhancement ratio
eGFR	Estimated glomerular filtration rate
EGPA	Eosinophilic granulomatosis with polyangiitis
EI	Eccentricity index
EL	Energy loss
ELBO	Evidence lower bound
Ell	Longitudinal strain
EMA	European Medicines Agency
Emax	Maximal end-systolic elastance
EMB	Endomyocardial biopsy
EMG	Electromyogram
EMI	Electromagnetic interference
EMS	Emergency medical services
ENDO	Endocardium/endocardial
ENMC	European Neuromuscular Centre
EOA	Effective orifice area
EOAI	Effective orifice area index
EP	Electrophysiological
*EPI*	*Echoplanar imaging*
*EPI*	*Epicardium/epicardial*
EQ	Energy quotient
EROA	Effective regurgitant orifice area
Err	Radial strain
ERS	European Respiratory Society
*ES*	*End-systole*
*ES*	*Edge sharpness*
*ES*	*Eisenmenger syndrome*
ESC	European Society of Cardiology
ESCR	European Society of Cardiovascular Radiology
ESD	End-systolic dimension
ESFS	End-systolic fiber stress
ERS	European Respiratory Society
ESMA	Elastin specific magnetic resonance agent
ESNR	Estimated signal-to-noise ratio
ESPVR	End-systolic pressure volume relationship
ESR	European Society of Radiology
ESRD	End-stage renal disease
ESS_sep_	End-systolic septal strain
ESV	End-systolic volume
ESVI	End-systolic volume index
ESWS	End-systolic wall stress
ETA	Elongated transverse aortic arch
ETL	Echo train length
EWA	Expectation maximization weighted algorithm
Ex-CMR	Exercise stress cardiovascular magnetic resonance
*FA*	*Flip angle*
*FA*	*Fatty acid*
*FA*	*Fractional anisotropy*
FAC	Fractional area change
FB	Free breathing
FBG	Fasting blood glucose
FC	Fibrous cap
FCN	Fully convolutional neural network
FCNN	Fully connected neural network
*FCR*	*Fibrous cap rupture*
*FCR*	*Flow convergence region*
FCSA	Fast composite splitting algorithm
FCW	Forward compression wave
*FD*	*Flow diverter*
*FD*	*Fractal dimension*
FDA	United States Food and Drug Administration
FE	Ferumoxytol enhanced
FED	Fibroelastic deficiency
FEV1	Forced expiratory volume
FID	Free induction decay
FDA	United States Food and Drug Administration
FDG	Fluorodeoxyglucose
FEV1	Forced expiratory volume in one second
FFE	Fast field echo
FFR	Fractional flow reserve
FFT	Fast Fourier transform
FFTO	Fontan fenestration test occlusion
FFV	Forward flow volume
FGP	Fast gradient projection
*FH*	*Foot-head*
*FH*	*Family history*
FHS	Framingham Heart Study
FIDDLE	Flow independent dark-blood delayed enhancement
FIRE	Framework for image reconstruction
FISTA	Fast iterative shrinkage-threshold algorithm
FL	False lumen
FLAIR	Fluid attenuated inversion recovery
FLASH	Fast low angle shot
FLEF	False lumen ejection fraction
FM	First order moment
fMRI	Functional magnetic resonance imaging
FN	False negative
fNAV	Focused navigation
FOV	Field-of-view
FP	False positive
FPP	First pass perfusion
FPR	False positive rate
*FS*	*Fat saturation*
*FS*	*Fractional shortening*
FSHD1	Facioscapulohumeral muscular dystrophy type 1
FSE	Fast spin echo
FSL	Spin lock frequency
*FT*	*Fourier transform*
*FT*	*Feature tracking*
FTAAD	Familial Thoracic Aortic aneurysms and dissection syndrome
FVC	Forced vital capacity
FW	Free wall
FWHM	Full width at half maximum
FWLS	Free wall longitudinal strain
GA	Gestational age
GAN	Generative adversarial network
GBCA	Gadolinium based contrast agent
GBM	Gradient boosting machine
GC-LOLA	Gradient controlled local Larmor adjustment
GCS	Global circumferential strain
GCSR	Global circumferential strain rate
Gd	Gadolinium
GDMT	Goal directed medical therapy
GFA	Generalized fractional anisotropy
GLCM	Gray-level co-occurrence matrix
GLRLM	Gray-level run-length matrix
GLM	General linear models
GLS	Global longitudinal strain
GLSR	Global longitudinal strain rate
GPAC	Global Physical Activity Questionnaire
GPU	Graphical processor units
GQI	Generalized Q-space imaging
Grad-CAM	Gradient-weighted class activation mapping
GRAPPA	Generalized autocalibrating partially parallel acquisition
GraSE	Gradient and spin echo
GRASP	Golden angle radial sparse parallel
GRE	Gradient recalled echo
GRS	Global radial strain
GS	Golden-step
GSS	Global severity score
GT	Ground truth
GWAS	Genome wide association study
H&E	Hematoxylin and eosin
HA	Helix angle
HARP	Harmonic phase magnetic resonance
HASTE	Half-Fourier single shot turbo spin echo
Hb	Hemoglobin
HbA1c	Hemoglobin A1c
HCM	Hypertrophic cardiomyopathy
Hct	Hematocrit
HCTsyn	Synthetic hematocrit
H_d_	Helical density
HD	Housdorff distance
HDL	High density lipoprotein
HDPE	High-density polyethylene
HE	Hematoxylin and eosin
HEIDI	Homogeneity-enabled incremental dipole inversion
HES	Hyperesoinophilic syndrome
HF	Heart failure
HFI	Helical flow index
HFmrEF	Heart failure with mid-range ejection fraction
HFpEF	Heart failure with preserved ejection fraction
HFrEF	Heart failure with reduced ejection fraction
Hb	Hemoglobin
HFR	Holodiastolic flow reversal
HHD	Hypertensive heart disease
HHFP	Hypertension-associated heart failure in pregnancy
HHV	Human herpes virus
HIP	High intensity plaque
HIV	Human immunodeficiency virus
HIVAC	Human immunodeficiency virus associated cardiomyopathy
HLA	Horizontal long axis
HLHS	Hypoplastic left heart syndrome
HOCM	Hypertrophic obstructive cardiomyopathy
HOMA-IR	Homeostasis model assessment-estimated insulin resistance
HP	Hyperpolarized
HPF	High-power field
HPLHS	Hypoplastic left heart syndrome
*HR*	*Heart rate*
*HR*	*Hazard ratio*
*HR*	High resolution
HR-VWI	High resolution vessel wall imaging
HRS	Heart Rhythm Society
HS	High salt
hsCRP	High sensitivity c-reactive protein
hs-cT	High sensitivity cardiac troponin
hs-cTnI	High sensitivity cardiac troponin I
hs-cTnT	High sensitivity cardiac troponin T
HSCT	Hematopoietic stem cell transplantation
HU	Hounsfield units
HV	Hepatic vein
HW	Heart weight
I/R	Ischemia/reperfusion
IAA	Infrarenal abdominal aorta
IAD	Intracranial artery dissection
*ICA*	*Internal carotid artery*
*ICA*	*Iodinated contrast agent*
*ICA*	Invasive coronary angiography
ICC	Intraclass correlation coefficient
ICD	Implanted cardiodefibrillator
*ICE*	*Intracardiac echocardiography*
*ICE*	*Image reconstruction environment*
ICM	Ischemic cardiomyopathy
iCMR	Invasive cardiovascular magnetic resonance
ICTP	Type I collagen C terminal telopeptide
ICU	Intensive care unit
IDI	Integrative discrimination index
IF	Immunofluorescence
IFG	Impaired fasting glucose
iFR	Instantaneous wave-free ratio
IFT	Inverse Fourier transform
IHC	Immunohistochemical
IHD	Ischemic heart disease
IHG	Isometric hand grip
IIM	Idiopathic inflammatory myopathy
ILT	Intraluminal thrombus
IMCL	Intramyocardial lipids
IMH	Intramyocardial hemorrhage
iNAV	Image-based navigator
INCA study	Impact of Non-invasive CMR Assessment
iNO	Inhaled nitric oxide
INOCA	Ischemia with no obstructive coronary arteries
INR	International normalized ratio
IO	Iron overload
IOC	Iron overload cardiomyopathy
IoU	Intersection over union
IPAH	Idiopathic pulmonary artery hypertension
IPH	Intraplaque hemorrhage
iPTH	Immunoreactive parathyroid hormone
IQ	Image quality
IQA	Image quality assessment
IQR	Interquartile range
IR	Inversion recovery
*IRF*	*Impulse response function*
*IRF*	*In-plane rotational flow*
IRSE	Inversion recovery spin echo
IRSF	Inversion recovery snapshot flash
ISF_sep-lat_	Internal stretch factor
ISHLT	International Society of Heart and Lung Transplantation
ISMRMRD	International Society for Magnetic Resonance in Medicine Raw Data
ISO	Isotropic diffusion component
IV	Intravenous
IVIG	Intravenous gamma immunoglobulin
IVMD	Interventricular mechanical delay
IVUS	Intravascular ultrasound
IVC	Inferior vena cava
IVS	Interventricular septum
IVST	Interventricular septal thickness
IVUS	Intravascular ultrasound
JSENSE	Joint image reconstruction and sensitivity estimation in sensitivity encoding
Kat-ARC	K-adaptive-t autocalibrating reconstruction for cartesian sampling
KD	Kawasaki disease
KE	Kinetic energy
KE_iEDV_	Kinetic energy normalized to left ventricular end-diastolic volume
Kt-BLAST	Kt broad linear speed up technique
L-L	Leading to leading
L-TGA	Levo-transposition of the great arties
*LA*	*Left atrium/left atrial*
*LA*	*Long axis*
*LA*	*Left anterior*
LAA	Left atrial appendage
Lac	Lactate
LAD	Left atrial descending coronary artery
LAEF	Left atrial emptying fraction
LAAEmF	Left atrial active emptying function
LAPEmF	Left atrial passive emptying function
LASSO	Least absolute shrinkage and selection operator
LATEmF	Left atrial total emptying function
LAV	Left atrial volume
LAVI	Left atrial volume index
LAVmax	Maximal left atrial volume
LAVmax-I	Maximal left atrial volume indexed to body surface area
LAVmin	Minimal left atrial volume
LAVmin-I	Minimal left atrial volume indexed to body surface area
LAx	Long axis
LBF	Lower body fat
LBBB	Left bundle branch block
LBP	Local binary patterns
LCA	Left coronary artery
LCBI	Lipid coreburden index
LCP	Leadless cardiac pacemaker
LCX	Left circumflex coronary artery
LDA	Linear discriminant analysis
LDH	Lactate dehydrogenase
LDL	Low density lipoprotein
LDS	Loeys-Dietz syndrome
LE	Loeffler’s endocarditis
LFP	Linear flip angle
LGE	Late gadolinium enhancement
LHC	Left heart catheterization
LHM	Left handed helix angle
LIPV	Left inferior pulmonary vain
LISA	Linearly increasing start-up angles
*LL*	*Lower limit*
*LL*	*Lower limb*
LLC	Lake Louise criteria
*LM*	*Left main coronary artery*
*LM*	*Loose matrix*
LMS	Lambda-Mu-Sigma
LNH	Local normalized helicity
LOA	Limits of agreement
LOE	Level of evidence
LOS	Length of stay
LOST	LOw-dimensional-structure Self-learning and Thresholding
*LP*	*Left posterior*
*LP*	*Label propagation*
LPA	Left pulmonary artery
*LR*	*Left–right*
*LR*	*Low resolution*
*LR*	*Logistic regression*
LRNC	Lipid rich necrotic core
LRP	Lipid rich plaque
LS	Longitudinal strain
LSCA	Left subclavian artery
LSPV	Left superior pulmonary vein
LT	Lateral tunnel
LV	Left ventricle/left ventricular
LVAD	Left ventricular assist device
LVEDVP	Left ventricular end-diastolic pressure
LVEDV	Left ventricular end-diastolic volume
LVEDVI	Left ventricular end-diastolic volume index
LVEF	Left ventricular ejection fraction
LVESV	Left ventricular end-systolic volume
LVM	Left ventricular mass
LVMI	Left ventricular mass index
LVMP	Left ventricular myocardial power
LVNC	Left ventricular non-compaction
LVOT	Left ventricular outflow tract
LVOTO	Left ventricular outflow tract obstruction
LVRR	Left ventricular reverse remodeling
LW	Linewidth
LWD	Lung water density
M1	Middle cerebral artery
M2	Second order motion compensation
*MA*	*Mitral annulus/mitral annular*
*MA*	*Methamphetamine-associated*
MA-CMP	Metamphetamine associated cardiomyopathy
mAA	Mid-ascending aorta
MAAD	Mid ascending aorta diameter
mAAr	Mid aortic arch
MAC	Moving angle crossing
MACE	Major adverse cardiovascular event
MAD	Mitral annular disjunction
MAE	Mean absolute error
mAoP	Mean aortic pressure
MAP	Mean arterial pressure
MAPE	Mean average percentage error
MAPK	Mitogen activated protein kinase
MAPSE	Mitral annular plane systolic excursion
MaR	Myocardium at risk
MARC	Markers And Response to CRT study
maxLCBI4mm	Maximum 4-mm lipid core burden index
MBF	Myocardial blood flow
MBG	Myocardial blush grade
MBP	Mean blood pressure
MBV	Myocardial blood volume
MCA	Middle cerebral artery
MCD	Mean contour distance
MCE	Myocardial contrast echocardiography
MCF	Myocardial contraction fraction
MCP	Monocyte chemoattractant protein
*MD*	*Mean diffusivity*
*MD*	*Muscular dystrophy*
MD2	Myotonic dystrophy II
MDCT	Multidetector computed tomography
MDIR	Multislice double inversion recovery
mDixon	Modified Dixon
mDA	Mid descending aorta
MDT	Mitral deceleration time
MEDI	Morphology enabled dipole inversion
MERGE	Motion sensitized driven equilibrium rapid gradient echo
MESA	Multi-Ethnic Study of Atherosclerosis
MESE	Multi-echo spin echo
MeSH	Medical Subject Heading
MET	Metabolic equivalent
MFA	Myocyte fractional anisotropy
MFR	Myocardial flow reserve
MFS	Marfan syndrome
MHD	Magnetohydrodynamic effect
MI	Myocardial infarction
MICSR	Magnitude image CSPAMM
MINOCA	Myocardial infarction with no obstructive coronary arteries
MIO	Myocardial iron overload
MIP	Maximal intensity projection
MIS	Multisystem inflammatory syndrome
MIS-C	Multisystem inflammatory syndrome in children
ML	Machine learning
MLHFQ	Minnesota Living with Heart Failure Questionnaire
mLV	Mid-left ventricle
mLVEF	Mid-range left ventricular ejection fraction
MM	Mitochondrial related mutation
MMD	Myotonic muscular dystrophy
MMP	Metalloproteinases
MMRC	Modified Medical Research Council
Mn	Manganese
MO	Microvascular obstruction
MOCO	Motion corrected
MOG	Metric optimized gating
MOLLI	MOdified Look Locker Inversion recovery
MOOSE	Meta-analysis Of Observational Studies in Epidemiology
MP	Myocardial perfusion
MP-RAGE	Magnetization prepared rapid acquisition gradient echo
MPA	Main pulmonary artery
mPAP	Mean pulmonary artery pressure
MPBF	Maldistribution of pulmonary blood flow
MPD	Maximum perpendicular distance
MPG	Mean pressure gradient
MPI	Myocardial perfusion imaging
MPO	Myeloperoxidase
*MPR*	*Myocardial perfusion reserve*
*MPR*	*Multiplanar reconstruction/reformatting*
MPRAGE	Magnetization prepared rapid acquisition gradient echo
MPRI	Myocardial perfusion reserve index
*MR*	*Magnetic resonance*
*MR*	*Mitral regurgitation*
MR-IMPACT	CMR for Myocardial Perfusion Assessment in Coronary Artery Disease
MR-INFORM	Magnetic Resonance Perfusion or Fractional Flow Researve in Coronary Artery Disease trial
MRA	Magnetic resonance angiography
mRAP	Mean right atrial pressure
MRE	Magnetic resonance elastography
MRegur	Mitral regurgitation
MRI	Magnetic resonance imaging
mRNA	Messenger RNA
MS	Mitral stenosis
MRS	Magnetic resonance spectroscopy
mSASHA	Modified saturation recovery single-shot acquisition
mSAX	Midventricular short axis
MSD	Mean surface distance
MSDR	Maximum systolic deceleration rate
MSE	Mean squared error
MSI	Myocardial salvage index
MT	Magnetization transfer
MTC	Magnetization transfer contrast
mtDNA	Mitochondrial DNA
MTG	Myocardial triglyceride content
MUGA	Multi-acquisition gated angiography
MUSIC	Multiphase steady-state imaging with contrast enhancement
*MV*	*Mitral valve*
*MV*	*Mixed venous*
MVA	Mitral valve area
MVD	Microvascular disease
MVO	Microvascular obstruction
MVO_2_	Myocardial oxygen consumption
MVP	Mitral valve prolapse
MVPA	Moderate to vigorous physical activity
*MVR*	*Mass volume ratio*
*MVR*	*Mitral valve repair*
*MVR*	Mitral valve replacement
MVV	Maximal voluntary ventilation
MWS	Mid-wall striae
MWT	Maximal wall thickness
MYO	Myohemoglobin
n-SD	Number of standard deviations
NASCET	North American Symptomatic Carotid Endarterectomy Trial
NAV	Navigator
NASCI	North American Society of Cardiovascular Imaging
*NC*	*Non-compacted*
*NC*	*Necrotic core*
*NC*	*Non-connective tissue*
*NC*	*Non-contrast*
NCS	Normalized circumferential strain
n.d	Non-dimensional
NDCM	Non-ischemic dilated cardiomyopathy
nDNA	Nuclear DNA
NF	Net flow
NFG	Non-fasting glucose
NHS	National Health Service
NICM	Non-ischemic cardiomyopathy
NHLBI	National Heart Lung and Blood Institute
NIHSS	National Institutes of Health Stroke Scale
NIRS	Near infrared spectroscopy
NIST	National Institute of Standards and Technology laboratory
NLP	Newborn Lung Project
NO	Nitric oxide
NOS	Newclastle-Ottawa quality assessment scale
NR	Non-rigid
nRDI	Non-restricted diffusion index
NRI	Net reclassification index
*NS*	*Non-selective*
*NS*	*Normal salt*
NSF	Nephrogenic systemic fibrosis
NSTEMI	Non ST elevation myocardial infarction
NSVT	Non-sustained ventricular tachycardia
NT-pro BNP	N-terminal pro-hormone brain natriuretic peptide
NWI	Normalized wall index
NYD	Not yet diagnosed
NYHA	New York Heart Association
O–O	Outer to outer
OCT	Optical coherence tomography
OPF	Orientation distribution function
OHCA	Out-of-hospital cardiac arrest
OMT	Optimal medical therapy
OR	Odds ratio
ORO	Oil red O
OS	Oxygen sensitive
OSA	Obstructive sleep apnea
OSI	Oscillatory shear index
OV	Overlapping
OVS	Outer volume suppression
OXPHOS	Oxidative phosphorylation
*PA*	*Popliteal artery*
*PA*	*Pulmonary artery*
PAB	Pulmonary artery banding
PAC	Pulmonary artery compliance
PACS	Picture archiving and communication system
PAD	Peripheral arterial disease
pAF	Paroxysmal atrial fibrillation
PAH	Pulmonary artery hypertension
PANDA	Principle component analysis and dictionary learning
PAP	Pulmonary artery pressure
PAPR	Powered air-purifying respirators
PAPVC	Partial anomalous pulmonary venous connection
PAPVR	Partial anomalous pulmonary venous return
PAQ-C	Physical Activity Questionnaire for older Children
PAS	Pulmonary artery stenosis
PASC	Post-acute sequelae Covid-19
PASP	Pulmonary artery systolic pressure
PAWP	Pulmonary artery wedge pressure
PAWS	Phase-ordered automatic window selection
PBV	Pulmonary blood volume
PBVV	Pulmonary blood volume variation
*PC*	*Phase contrast*
*PC*	*Principle component*
*PCA*	*Principal component analysis*
*PCA*	*Phase contrast angiography*
PCI	Percutaneous coronary intervention
PCMR	Phase contrast magnetic resonance
PCr	Phosphocreatine
PCR	Polymerace chain reaction
PCS	Peak circumferential strain
*PDA*	*Patent ductus arteriosus*
*PDA*	*Posterior descending coronary artery*
*PDF*	*Projection onto dipole fields*
*PDF*	*Probability distribution function*
PDFF	Proton density fat fraction
PDGF	Platelet derived growth factor
PDSRC	Peak diastolic circumferential strain rate
PDSRL	Peak diastolic longitudinal strain rate
PDSRR	Peak diastolic radial strain rate
PDw	Proton density weighted
PCWP	Pulmonary capillary wedge pressure
PDH	Pyruvate dehydrogenase
*PE*	*Phase encoding*
*PE*	*Potential energy*
*PE*	*Pulmonary embolism*
*PE*	*Parameter estimates*
*PEA*	*Pulseless electrical activity*
*PEA*	*Pulmonary endarterectomy*
PET	Positron emission tomography
PF	Peak flow
PFA	Perfluoroalkoxyalkane
*PFR*	*Peak filling rate*
*PFR*	*Perivascular fibrosis*
PG	Pressure gradient
PGSE	Pulse gradient spin echo
PH	Pulmonary hypertension
PHiSeg	Probabilistic hierarchical segmentation
*PHT*	*Pediatric heart transplantation*
*PHT*	*Pressure half-time*
PI	Pulsatility index
PICA	Posterior inferior cerebral artery
PICS	Parallel imaging compressed sensing
PISA	Proximal isovelocity surface area
PLAX	Parasternal long axis
PLM	Polarized light microscopy
pLVEF	Preserved left ventricular ejection fraction
PLS	Peak longitudinal strain
PLSVC	Persistent left superior vena cava
PM	Papillary muscle
pMI	Periprocedure myocardial injury
PML	Posterior mitral leaflet
PMMA	Polymethyl methacrylate
PMR	Plaque to myocardial signal intensity ratio
PNF	Pulmonary net flow
POC	Point-of-care
POMP	Phase offset multiplanar
PP	Pulse pressure
PPE	Personal protective equipment
PPCI	Primary percutaneous coronary intervention
PPCM	Peripartum cardiomyopathy
PPG	Peak pressure gradient
PPI	Pitch per inch
PPM	Permanent pacemaker
*PR*	*Precision recall*
*PR*	*Pulmonic regurgitation*
PR%	Pulmonary regurgitation fraction
Prec	Precision
PRESS	Point resolved spectroscopy
PRF	Pulmonary regurgitant fraction
PRISM	Preferred reporting items for systemic reviews and meta-analysis
PRISMA	Preferred reporting items for systematic reviews and meta analyses
PROST	Patch-based low-rank reconstruction
PROUD	Prospective undersampling in multiple dimensions
PRS	Peak radial strain
PRV	Pulmonary regurgitant volume
PRVI	Pulmonary regurgitant volume index
pSAT	Partial saturation
PSF	Point spread function
PSAX	Parasternal short axis
PSIR	Phase sensitive inversion recovery
PSM	Propensity score matching
P_SYS_	Peak systolic pressure
PSSR	Peak systolic strain rate
PSSRC	Peak systolic circumferential strain rate
PSSRL	Peak systolic longitudinal strain rate
PSSRR	Peak systolic radial strain rate
PTB	Pulmonary transit beats
PTH	Parathyroid hormone
PTT	Pulmonary transit time
PTFE	Polytetrafluoroethylene
*PV*	*Pulmonary valve*
*PV*	*Pulmonary vein*
*PV*	*Pressure volume*
*PV*	*Peak velocity*
PVA	Pulmonary valve annulus
*PVC*	*Polyvinyl chloride*
*PVC*	*Premature ventricular complexes*
PVDR	Pulmonary vascular distensibility reserve
PVI	Pulmonary vein isolation
PVL	Paravalvular leak
PVO_2_	Peak oxygen comsumption
PVOD	Pulmonary veno-occlusive disease
*PVR*	Pulmonic valve replacement
*PVR*	Pulmonary vascular resistance
PVRI	Pulmonary vascular resistance index
PWV	Pulse wave velocity
Pyr	Pyruvate
Q	Flow
QALY	Quality-adjusted life year
QC	Quality control
QCA	Quantitative coronary angiography
QIBA	Quantitative Imaging and Biomarkers Alliance
QIR	Quadruple inversion recovery
QISS	Quiescent interval slice-selective
QoL	Quality of life
Qp	Pulmonic flow
Qs	Systemic flow
QSM	Quantitative susceptibility mapping
QTc	Corrected QT interval
*RA*	*Right atrium/right atrial*
*RA*	*Right-anterior*
*RAA*	*Right aortic arch*
*RAA*	*Right atrial appendage*
RAEF	Right atrial emptying fraction
RAC	Relative area change
rAHP	Reverse adiabatic half passage
RAP	Right atrial pressure
RARE	Rapid acquisition with relaxation enhancement
RAPID-IHD	Rapid Cardiovascular Magnetic Resonance for Ischemic Heart Disease
RAS	Renin-angiotensin system
RAV	Right atrial volume
RAVI	Right atrial volume index
R_BP_	Mean radius of the blood pool
rBW	Receiver bandwidth
*RCA*	*Right coronary artery*
*RCA*	*Reverse classification accuracy*
RCO	Right coronary ostium
RCT	Randomized controlled trial(s)
RDI	Restricted diffusion index
REACT	Relaxation-enhanced angiography without contrast and triggering
Rec	Recall
ReLU	Rectified linear unit
RER	Respiratory exchange ratio
*RF*	*Radiofrequency*
*RF*	*Regurgitant fraction*
*RF*	*Random forests*
RF2	Random Forests
RHC	Right heart catheterization
RHM	Right handed orientation
RIPV	Right inferior pulmonary vein
RL	Right-left
rLVEF	Reduced left ventricular ejection fraction
RMPV	Right middle pulmonary vein
RMS	Root mean square
RMSD	Root mean square distance
RMSE	Root mean square error
*ROC*	*Receiver operator characteristics*
*ROC*	*Receiver operator curve*
ROI	Region-of-interest
ROS	Reactive oxygen species
RP	Right posterior
*RPA*	*Recursive partitioning analysis*
*RPA*	*Right pulmonary artery*
RPP	Rate pressure product
*RRT*	*Renal replacement therapy*
*RRT*	*Relative residence time*
*RS*	*Radial strain*
*RS*	*Rejection score*
RSD	Relative standard deviation
RSN	Radial self-navigated
RSNA	Radiological Society of North America
RSPV	Right superior pulmonary vein
RT	Real time
RT-PCR	Reverse transcription-polymerase chain reaction
RTC	Real time cine
rTOF	Repaired tetralogy of Fallot
RTP	Return to play
RU	Relative upslope
*RV*	*Right ventricle/right ventricular*
*RV*	*Regurgitant volume*
RVD	Right ventricular dilation
RVED	Right-ventricular end-diastolic volume
RVEDVI	Right ventricular end-diastolic volume index
RVEF	Right ventricular ejection fraction
RVESVI	Right ventricular end-systolic volume index
RVFW	Right ventricular free wall
RVFWS	Right ventricular free wall strain
RVH	Right ventricular hypertrophy
RVI	Right ventricular insertion
RVLA	Right ventricular long axis
RVol	Regurgitant volume
RVOT	Right ventricular outflow tract
RVSP	Right ventricular systolic pressure
RVT	Retrospective valve tracking
RWM	Regional wall motion
RWMA	Regional wall motion abnormality
RWT	Relative wall thickness
S-ICD	Subcutaneous implantable cardioverter defibrillator
SAA	Serum amyloid A
SAEs	Serious adverse events
SALLI	Small animal Look Locker inversion recovery
SAM	Systolic anterior motion
SAPPHIRE	Saturation pulse prepared heart rate independent inversion recovery
SAR	Specific absorption rate
SARS-CoV-2	Severe acute respiratory syndrome coronavirus 2
SASHA	Saturation recovery single-shot acquisition
*SAT*	*Saturation pulse*
*SAT*	*Subcutaneous adipose tissue*
SAVR	Surgical aortic valve replacement
SAx	Short axis
SBP	Systolic blood pressure
SCAI	Society for Cardiovascular Angiography and Interventions
SCCT	Society of Cardiovascular Computed Tomography
SCD	Sudden cardiac death
SCMR	Society for Cardiovascular Magnetic Resonance
SCS	Systolic circumferential strain
SD	Standard deviation
SDp	Pooled standard deviation
SE	Spin echo
SEE	Standard error of the estimate
SENC	Strain encoding
SENSE	Sensitivity encoding
SFA	Superficial femoral artery
SFRR	Systolic flow reversal ratio
SFT	Semi-automated flow tracking
SG	Self-gated
*SIR*	*Stress-to-rest intensity ratio*
*SIR*	*Selective inversion recovery*
*SIR*	*Signal intensity ratio*
SIT	Situs inversus totalis
sGS	Sorted golden step
ShMOLLI	Shortened modified Look Locker inversion recovery
*SI*	*Signal intensity*
*SI*	*Superior–inferior*
SIP	Septal insertion points
SIR	Signal intensity ratio
SL	Spin lock/spin locking
sLASER	Semi-adiabatic localization by adiabatic selective refocusing
SLE	Systemic lupus erythematosus
SLFF	Semilunar valve forward flow
SLICE	Segment length in cine
SLNF	Semilunar valve net flow
SLS	Segmental longitudinal strain
SLV	Single left ventricle
*SM*	*Sarcomere mutation*
*SM*	*Shape mode*
SMA	Superior mesenteric artery
SMASH	Simultaneous acquisition of spatial harmonics
SMC	Smooth muscle cells
SMD	Standardized mean difference
SMR	Spleen-to-myocardium ratio
SMS	Simultaneous multi-slice
SN	Self-navigated
SNA	Sympathetic nerve activity
SNAP	Simultaneous non-contrast angiography and intraplaque hemorrhage
SNR	Signal-to-noise ratio
SO	Second observer
SOP	Standard operating procedures
SOS	Stack of stars
SP	Sinus prosthesis
SPACE	Sampling perfection with application-optimized contrast using different flip angle evolutions
SPAIR	Spectral attenuated inversion recovery
sPAP	Systolic pulmonary artery pressure
SPCTPD	Society of Pediatric Cardiology Training Program Directors
SPAMM	Spatial modulation of magnetization
SPAIR	Spectral attenuated inversion recovery
SPECT	Single photon emission computed tomography
SPGR	Spoiled gradient echo
SPINS	Stress CMR Perfusion Imaging in the United States
SPIO	Small particle iron oxide
SPIR	Spectral presaturation with inversion recovery
SPR	Splenic perfusion ratio
SQ	Semi-quantitative
*SR*	*Strain rate*
*SR*	*Sinus rhythm*
*SR*	*Super resolution*
*SR*	*Saturation recovery*
SRR	Super resolution reconstruction
SRS	Segmental radial strain
SRS_sep_	Systolic rebound stretch of the septum
SRV	Single right ventricle
SS	Slice-selective
SSc	Systemic sclerosis
SSDI	Social Security Death Index
SSFP	Steady state free precession
SSH	Secure shell protocol
SSI	Systolic stretch index
SSIM	Structured similarity index
SScPAH	Systemic sclerosis pulmonary artery hypertension
bSSFP	Balanced steady state free precession
SSO	Splenic switch-off
SSR	Single volume super-resolution reconstruction
SSTSE	Single shot turbo spin echo
STE	Speckle tracking echocardiography
STEAM	Stimulated echo acquisition mode
STEMI	ST elevation myocardial infarction
STI	Susceptibility tensor imaging
STIR	Short tau inversion recovery
STJ	Sinotubular junction
STRM	Signal threshold versus reference mean
STS	Surgical Thoracic Society
*SV*	*Stroke volume*
*SV*	*Single ventricle*
SVC	Superior vena cava
SVD	Single value decomposition
SVE	Shared velocity encoding
SVI	Stroke volume index
SVM	Support vector machines
*SVR*	*Systemic vascular resistance*
*SVR*	*Slice-to-volume registration*
SVT	Supraventricular tachycardia
SW	Stroke work
S_WALL_	Mean myocardial wall thickness
T	Tesla
T1DM	Type 1 diabetes mellitus
T1w	T1 weighted
T2DM	Type 2 diabetes mellitus
T2prep	T2 preparation
T2w	T2 weighted
*TA*	*Transverse angle*
*TA*	*Texture analysis*
*TA*	*Tricuspid annulus/tricuspid annular*
*TA*	*Tricuspid atresia*
TA-WSS	Time averaged wall shear stress
*TAC*	*Total arterial compliance*
*TAC*	*Transverse aortic constriction*
*TAC*	*Thoracic aortic calcification*
TACi	Total arterial compliance index
TAO	Transverse aortic arch
TAPSE	Tricuspid annular plane systolic excursion
TAPVC	Total anomalous pulmonary vein connection
TAV	Trileaflet aortic valve
TAVI	Transcatheter aortic valve implantation
TAVR	Transcatheter aortic valve replacement
TB	Tuberculosis
TBAD	Type B aortic dissection
TCFA	Thin-cap fibroatheroma
TCM	Takotsubo cardiomyopathy
TCPC	Total cavopulmonary connection
*TD*	*Delay time*
*TD*	*Time difference*
*TD*	*Trigger delay*
TDI	Tissue Doppler imaging
TE	Echo time
TE_eff_	Effective echo time
TEE	Transesophageal echocardiography
TEM	Transmit-receive electromagnetic
TEVAR	Thoracic endovascular aortic repair
TFC	Task Force Criteria
TFE	Turbo field echo
TG	Triglyceride
TGA	Transposition of the great arteries
TGF-*B*1	Transforming growth factor beta-1
THR	Target heart rate
TI	Inversion time
TIA	Transient ischemic attack
TIMI	Thrombolysis in myocardial infarction
TIMP	Tissue inhibitors of matrix metalloproteinases
TIO	Transfusion iron overload
TKE	Turbulent kinetic energy
TL	True lumen
TM	Mixing time
*TMA*	*Trimethylammonium*
*TMA*	*Trimethyl amide*
TN	True negative
TOF	Tetralogy of Fallot
TOST	Two-sided test of equivalence
*TP*	*True positive*
*TP*	*Tube prosthesis*
TPG	Transpulmonary pressure gradient
*TPM*	*Tissue phase mapping*
*TPM*	*Trabeculae and papillary muscles*
*TPR*	*True positive rate*
*TPR*	*Total pulmonary resistance*
*TR*	*Repetition time*
*TR*	*Tricuspid regurgitation*
*TR*	*Time resolved*
TRAMINER	Transfer and inversion recovery-prepared imaging
TS	Saturation delay
TSE	Turbo spin echo
TSI	Time signal intensity
TSL	Spin lock time
TT	Transit time
TTC	Triphenyltetrazolium chloride
TTE	Transthoracic echocardiography
*TV*	*Total variation*
*TV*	*Tricuspid valve*
TVD	Triple vessel disease
TVI	Time velocity integral
TxREF	Transmitter B1 reference
UAP	Unstable angina pectoris
UAV	Unicuspid aortic valve
UFA	Unsaturated fatty acid
UKBB	United Kingdom BioBank
*UL*	*Upper limit*
*UL*	*Upper limb*
ULN	Upper limits of normal
US	Ultrasound
USPIO	Ultrasmall particles of iron oxide
UTE	Ultrashort echo time
UV	Umbilical vein
UWDRS	Unified Wilson’s Disease Rating Scale
*VA*	*Ventricular arrhythmias*
*VA*	*Vertebral artery*
VAC	Ventricular arterial coupling
VAPOR	Variable pulse power and optimized relaxation
VAT	Visceral adipose tissue
VC	Vena contracta
VCAM	Vascular cell adhesion molecule
VCG	Vector electrocardiogram
VCO_2_	Carbon dioxide production
VD	Variable density
VD-CASPR	Variable density Cartesian trajectory with spiral profile
*VE*	*Ventilator efficiency*
*VE*	*Minute ventilation*
VEL	Viscous energy loss
VELR	Viscous energy loss rate
VENC	Velocity encoded
VES	Ventricular extra-systoles
VF	Ventricular fibrillation
VFA	Variable flip angle
VHA	Vena hemiazygos
VHD	Valvular heart disease
VIBE	Volumetric-interpolated breath-hold examination
VIP	Ventricular insertion points
VIPR	Isotropic voxel radial projection imaging
VLA	Vertical long axis
Vmax	Maximal velocity
VNR	Velocity to noise
VO_2_	Oxygen consumption
VOI	Volume of interest
VOL	Volume
Vp	Propagation velocity
VPS	Visual presence score
VQ	Vorticity quotient
VR	Volume rendered
vSaO_2_	Mixed venous oxygen saturation
VSARR	Valve sparing aortic root replacement
VSD	Ventricular septal defect
VSMC	Vascular smooth muscle cells
*VT*	*Ventricular tachycardia*
*VT*	*Ventilator threshold*
VTE	Venous thromboembolism
VTI	Vertebral tortuosity index
VTS	Visual transmurality score
VUS	Variant of uncertain significance
VV	Interventricular
Vwall	Myocardial wall volume
vWERP	Virtual work-energy relative pressure
VWI	Vessel wall imaging
wb-LGE	Wide band late gadolinium enhancement
WC	Waist circumference
WD	Wilson Disease
WE	Water excitation
WET	Water suppression enhanced through T1 effects
WH	Whole heart
WHO	World Health Organization
WHR	Waist hip ratio
WHtR	Waist to height ratio
WIA	Wave intensity analysis
WIP	Work in progress
WISE	Women Ischemia Syndrome Evaluation
WM	Wall motion
WMA	Wall motion abnormality
WMSI	Wall motion score index
WS	Wall stress
WSS	Wall shear stress
*WT*	*Wall thickness*
*WT*	*Wild type*
WU	Wood units
XA	X-ray angiography
XD-GRASP	Extradimensional golden-angle radial sparse parallel
XMR	Combined x-ray cardiac magnetic resonance laboratories
Zva	Valvuloarterial impedance

Multiuse abbreviations are displayed in italics text

Italics refers to abbreviations that may have multiple meanings (but only one in any single manuscript)

I encourage authors to carefully consider the number of significant digits and reported p values in their manuscripts. For example, when reporting native T1 and standard deviation, would report to the nearest ms and not to the X.X ms or X.XX ms. While technically accurate, reporting T1 to this level of accuracy has no clinical relevance. Similarly, when reporting p values for the sample sizes of most *JCMR* publications, a value of < 0.001 is a reasonable limit.

All work submitted to the *JCMR* must be original and *cannot be under consideration by another journal until a decision is made by the JCMR*. Though a rare occurrence, we have encountered instances where authors had multiple simultaneous submissions. When we become aware of this, the manuscript is immediately withdrawn from further consideration and the authors are put on administrative warning.

## Reviewer recognition—gold star reviewers

Reviewers are a key component to the success of the *JCMR*. In 2019, we introduced the annual JCMR Gold Star Reviewer recognition program for all those who had (1) reviewed at least 3 manuscripts (2) provided an on-time review and (3) provided a high quality review. For 2021, we also recognized the first *JCMR* Triple Gold Star Reviewer recognition for those who had received a gold start for 3 consecutive years. The 100 *JCMR* Gold Star reviewers and inaugural 31 JCMR Triple Gold Star reviewers are listed in Table 4. Please join the ranks of *JCMR* reviewers and strive to be a Gold Star reviewer! As an added incentive, reviewers have the option to receive continuing medical education (CME) credit for providing a review.

**Table 4:** 2021 JCMR gold star and **triple gold star reviewers**

Bradley D Allen
**Ryan Avery**
**Adrianus J. Bakermans**
W. Patricia Bandettini
Tamer Basha
Nicoleta Baxan
Giovanni Biglino
Kenneth Bilchick
David Alan Bluemke
Paco Bravo
**Andrea Cardona**
Marcus Carlsson
**YuCheng Chen**
Henry Chubb
Otavio Coelho-Filho
Francisco Contijoch
Ibolya Csecs
Francesca Nesta Delling
Jonas Doerner
Robert R. Edelman
**Michael Elliott**
Daniel Ennis
**Emil Knut Stenersen Espe**
**Ahmed Fahmy**
Zhaoyang Fan
Vanessa Melanie Ferreira
Christopher J Francois
Marco Francone
Jérôme Garot
Matthias Gero Friedrich
**Lindsay Griffin**
**Lars Grosse-Wortmann**
**Ying Kun Guo**
Reza Hajhosseiny
**Hassan Haji-Valizadeh**
Ahmed Hamimi
Ruud B van Heeswijk
**Markus Henningsson**
**Lazaro Eduardo Hernandez**
Kan N Hor
Andrew Howarth
**Peng Hu**
Edward Hulten
El-Sayed Ibrahim
Masaki Ishida
**Tevfik F Ismail**
Jason Nathaniel Johnson
Alexandros Kallifatidis
Shingo Kato
**Won Yong Kim**
**Grigorios Korosoglou**
**Ramkumar Krishnamurty**
Selcuk Kucukseymen
Andreas Kumar
**Deborah Kwon**
Seung-Pyo Lee
Simon Lee
Yue-Hin Loke
Massimo Lombardi
**Minjie Lu**
Julian Luetkens
Wojciech Mazur
Daniel R Messroghli
Lorenzo Monti
Kai Muellerleile
Vivek Muthurangu
Takeru Nabeta
AV Naumova
Muhummad Sohaib Nazir
**Thomas Neuberger**
Ming-Yen Ng
Christopher Nguyen
**Laura Olivieri**
Ellen Ostenfeld
**Dana Peters**
Arno Roest
**Tobias Rutz**
**Hajime Sakuma**
Michal Schafer
**Dipan J. Shah**
**Sujata M Shanbhag**
Chetan Shenoy
Orlando P. Simonetti
Timothy Slesnick
Sahar Soleimani
Jonathan Soslow
Pascal Spincemaille
**Monvadi Barbara Srichai-Parsia**
Jordan B. Strom
Michael D. Taylor
Robert Tunks
Yining Wang
Mark Westwood
John Wood
Lian-Ming Wu
**Yibin Xie**
**Alistair Young**
Karolina M Zareba
**Chengcheng Zhu**

Triple gold start reviewers are **bolded**

## Conflict-of-interest, reviews, SCMR guideline/position manuscripts and SCMR committee papers

Conflict-of-interest manuscripts, those for which a member of the associate editorial board is either an author, acknowledged in the manuscript or closely associated with an author, are independently handled by a Guest Editor (Table 5) chosen by me. Neither I nor any of the associate editorial board are involved with reviewer selection or with manuscript decision. Our managing editorial office assists the Guest Editor with the administrative software/Editorial Manager. If a conflict-of-interest manuscript is accepted, the Guest Editor is recognized in the *JCMR* publication with the text “Dr. XX served as a *JCMR* Guest Editor for this manuscript.”

**Table 5:** 2021 JCMR guest editors

Gerard Aurigemma
David Bluemke
Raymond Chan
Robert Edelman
Paul Finn
Robert Judd
Raymond Kim
Raymond Kwong
Joao A. C. Lima
Vivek Muthurangu
Laura Olivieri
Ellen Ostenfield
Nathaniel Reichek
Hajime Sakuma
Matthias Stuber
Robert Weiss

The *JCMR* does not accept unsolicited reviews. Authors are encouraged to contact the editor-in-chief (jcmreditor@scmr.org) before submitting any reviews. In general, we expect reviews to be authored by individuals considered experts in the field and receive considerable attention/downloads. All solicited reviews follow the usual peer-review process. Several reviews were published in 2021, including reviews on 4D flow in tetralogy of Fallot [10], dark blood CMR techniques [11], and COVID [12].

The *JCMR* is the official publication of the SCMR. As such, SCMR Guidelines and Position papers endorsed by the Full (or Executive) SCMR Board(s) do *not* undergo peer review. I review these manuscripts for consistency with *JCMR* style and abbreviations. They are then published in an expeditious manner. Society position papers included documents on Level II/independent practitioner training guidelines [12], writing standards for guidelines [13] and SCMR position paper on the role of CMR in women [14].

## SCMR case of the week series

While the *JCMR* does not accept case reports, for many years, the SCMR web site has an active “Case of the Week” (https://scmr.org/page/caseoftheweekLDGPG) series, currently coordinated by Dr. Sylvia Chen. For the second time, in 2021, we published the prior year’s annual case series as a single manuscript [15]. This unified publication is planned as an annual occurrence in *JCMR* to allow for these illustrative cases to be more widely available to search engines.

## Continuing medical education (CME) *JCMR* journal club

For over 4 years we have been offering on-line CME credit for the benefit of our clinician readers and is a free benefit for SCMR members -allowing them to more easily fulfill the CME criteria for maintenance of their Level II or III certification [16]. This program has been a great success and was greatly expanded with 14 manuscripts in 2021. (Table 6). Please see http://scmr.peachnewmedia.com/store/provider/custompage.php?pageid=20 for the complete listing.

**Table 6:** 2021 JCMR manuscripts chosen for continuing medical education (CME)

Theo Pezel	Prognostic value of stress cardiovascular magnetic resonance in asymptomatic patients with known coronary artery disease [17]
Claire E. Raphael	**CMR predictors of heart failure in hypertrophic cardiomyopathy: the role of myocardial replacement fibrosis and microcirculation** [24]
Thu-Thao Le	**Multiparametric exercise stress cardiovascular magnetic resonance in the diagnosis of coronary artery disease: the EMPIRE trial** [26]
Yvonne J.M. van Cauteren	Cardiovascular magnetic resonance accurately detects obstructive coronary artery disease in suspected non-ST elevation myocardial infarction: a sub-analysis of the CARMENTA Trial [34]
David Marlevi	False lumen pressure estimation in type B aortic dissection using 4D flow cardiovascular magnetic resonance: comparisons with aortic growth [35]
Theo Pezel	**Long-term prognostic value of stress perfusion cardiovascular magnetic resonance in patients without known coronary artery disease** [25]
Reza Hajhosseiny	Clinical comparison of sub-mm high-resolution non-contrast coronary CMR angiography against coronary CT angiography in patients with low-intermediate risk of coronary artery disease: a single center trial [36]
Satoshi Nakamura	Long-term prognostic value of whole-heart coronary magnetic resonance angiography [37]
Ying Zhang	**Comparing cardiovascular magnetic resonance strain software packages by their abilities to discriminate outcomes in patients with heart failure with preserved ejection fraction** [27]
Aakash N. Gupta	Direct mitral regurgitation quantification in hypertrophic cardiomyopathy using 4D flow CMR jet tracking: evaluation in comparison to conventional CMR [38]
Luuk H.G.A. Hopman	Impaired left atrial reservoir and conduit strain in patients with atrial fibrillation and extensive left atrial fibrosis [39]
Andrew N. Jordan	Morphological and functional cardiac consequences of rapid hypertension treatment: a cohort study [40]
Shingo Kato	Prognostic value of resting coronary sinus flow determined by phase-contrast cine cardiovascular magnetic resonance in patients with known or suspected coronary artery disease [41]
Alastair J. Rankin	Myocardial changes on 3T cardiovascular magnetic resonance imaging in response to haemodialysis with fluid removal [42]

Bold manuscripts were also selected for 2021 JCMR Journal Club presentations

## Social media

I am very much a social media novice, but the *JCMR* continues to be very active on Twitter with the handle “JournalofCMR.” Tweets go out with the publication of each manuscript publication and announcing each Journal Club. This activity is coordinated by our two Social Media editors, Drs. Juan Lopez-Mattei and Purvi Parwani.

## Gerald M. Pohost and Dudley Pennell awards

In recognition of the efforts of our inaugural editor-in-chief, Dr. Gerald M. Pohost, (Fig. 3) for the past 15 years, the *JCMR* has awarded the Pohost Prize to that manuscript deemed by the associate editors and editorial board to be the best/most important manuscript published in the prior year. The associate editors and I select the Pohost finalists (Table 7) and the entire editorial board votes on the top prize. At the virtual 2021 SCMR Scientific Sessions annual meeting, the 15th Gerald M. Pohost Prize was awarded to Dr. Theo Pezel and co-workers for their manuscript “Prognostic value of vasodilator stress perfusion cardiovascular magnetic resonance after inconclusive stress testing.” [17]. The Pohost Runner-up Prize was awarded to Dr. Angelica Romero Daza and colleagues for their publication, “Mitral valve prolapse multifunctional features by cardiovascular magnetic resonance: more than just a valvular disease” [18].

**Table 7:** 2022 Gerald M. Pohost Award Finalists. Dr. Pezel [17] was the recipient of the 14th Gerald M. Pohost Award. Dr. Romero Daza [18] was the runner-up

Edelman, R.R., Leloudas, N., Pang, J. et al. Dark blood cardiovascular magnetic resonance of the heart, great vessels, and lungs using electrocardiographic-gated three-dimensional unbalanced steady-state free precession [31]
Edy, E., Rankin, A.J., Lees, J.S. et al. Cardiovascular magnetic resonance for the detection of descending thoracic aorta calcification in patients with end-stage renal disease [43]
Li, S., He, J., Xu, J. et al. Patients who do not fulfill criteria for hypertrophic cardiomyopathy but have unexplained giant T-wave inversion: a cardiovascular magnetic resonance mid-term follow-up study [44]
Loke, YH., Capuano, F., Cleveland, V. et al. Moving beyond size: vorticity and energy loss are correlated with right ventricular dysfunction and exercise intolerance in repaired Tetralogy of Fallot [45]
Nakamura, S., Ishida, M., Nakata, K. et al. Long-term prognostic value of whole-heart coronary magnetic resonance angiography [37]
Pezel, T., Unterseeh, T., Garot, P. et al. Prognostic value of vasodilator stress perfusion cardiovascular magnetic resonance after inconclusive stress testing [33]
Romero Daza, A., Chokshi, A., Pardo, P. et al. Mitral valve prolapse morphofunctional features by cardiovascular magnetic resonance: more than just a valvular disease [18]
Seraphim, A., Knott, K.D., Beirne, AM. et al. Use of quantitative cardiovascular magnetic resonance myocardial perfusion mapping for characterization of ischemia in patients with left internal mammary coronary artery bypass grafts [46]
Thompson, E.W., Kamesh Iyer, S., Solomon, M.P. et al. Endogenous T1ρ cardiovascular magnetic resonance in hypertrophic cardiomyopathy [47]
Zghaib, T., Te Riele, A.S.J.M., James, C.A. et al. Left ventricular fibro-fatty replacement in arrhythmogenic right ventricular dysplasia/cardiomyopathy: prevalence, patterns, and association with arrhythmias [48]

At that virtual meeting, we also presented the 4th Dudley Pennell Award in recognition of the foresight of *JCMR*’s 2nd Editor-in-Chief, Professor Dudley J. Pennell (Fig. 3) to transition the *JCMR* to the open-access platform (a decision (spearheaded by then SCMR Publications Committee chairman, Dr. Matthias Friedrich). Their decision markedly improved *JCMR*’s visibility and impact factor. The Pennell award is for that *original manuscript* that has most contributed to the *Journal’s* impact factor for the calendar year 3 years prior to the award. The 3rd Dudley J. Pennell Prize was awarded to Dr. Wenjia Bai et al. for their publication, “Automated cardiovascular magnetic resonance image analysis with fully convolutional networks” [19] with the runner-up Pennell Award was given to Dr. José Fernando Rodríguez -Palomares and colleagues for publication, “Aortic flow patterns and wall shear stress maps by 4D-flow cardiovascular magnetic resonance in the assessment of aortic dilation in bicuspid aortic valve disease” [20].

Stay tuned for the 15th Pohost and 4th Pennell Awards that will presented at the 23nd Scientific Sessions of the *Society* this February in Ft Lauderdale, Florida, USA!

## Tribute to Nathaniel Reichek

Last year the SCMR and the greater CMR community lost one of our founding fathers. Dr. Nathaniel Reichek. Nat was a friend and a true giant in our field. He was literally “in the room” when the SCMR was founded, served as our 3rd president, was a 2017 recipient of the SCMR Gold Medal, and was a tireless advocate for the United States CMR Advocacy Committee. Last year, the SCMR named the Education and Research Fund in his honor. For my tenure as editor-in-chief, Nat was often my “go to” person for conflict-of-interest manuscripts or sounding board. His command of CMR was almost unparalleled, and he readily gave his time to help the *Journal* and all who inquired of his opinion. While we didn’t agree on every issue, Nat was a gentleman of high integrity and I miss him at multiple levels. We published our first “In Memoriam” in his honor [21]. May his memory be a blessing.

## BMC publisher

For the past 15 years, the *JCMR* has been published by BMC, part of Springer Nature and a pioneer of open access publishing. Our current five-year contract with BMC ends at the end of 2022 and the SCMR has embarked on a search for a publisher (may remain with BMC but yet to be determined). Our new editor-in-chief, Tim Leiner is the chair of the committee and an RFP was recently distributed. A decision is expected by mid 2023. Regardless, the *Journal of Cardiovascular Magnetic Resonance, JCMR* moniker, and *Journal* contents are owned by the Society. The transition to a new publisher (if this occurs) at the end of 2023 will be seamless to you, our readership.

## Manuscripts—WordCloud

**Fig. 5:**
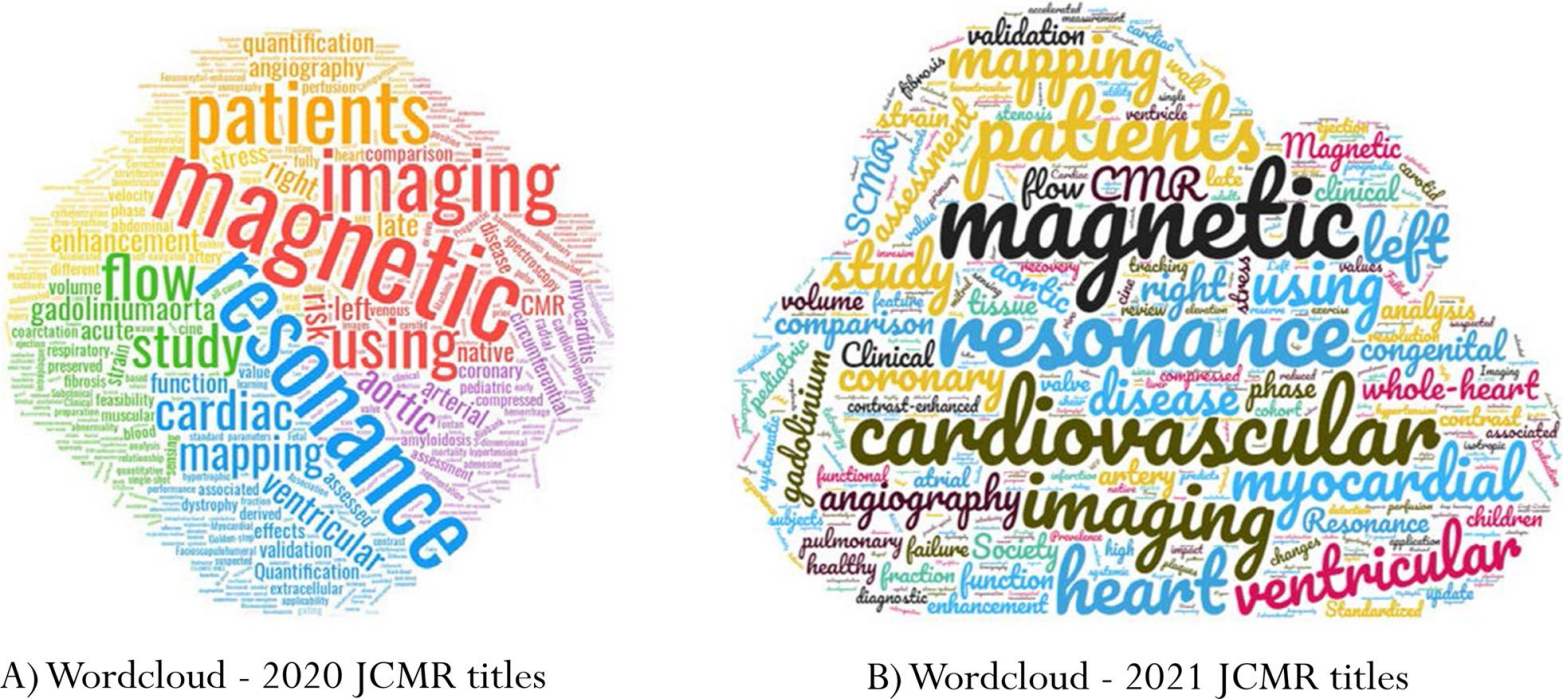
WordCloud of (**A**) 2020 and (**B**) 2021 *JCMR* manuscript titles

As in last year’s review, I chose to create a Wordcloud (https://www.wordclouds.com) of the 2020 and 2021 *JCMR* titles (Fig. 5). As in 2020, the most common JCMR manuscript title words were magnetic, cardiovascular, resonance with 2021 followed by imaging, heart, ventricular and myocardial.

I hope you have found my closing annual “State of our *JCMR*” informative. I remain the captain until December 31, 2022, but as members of the *SCMR*, it is really your *Journal* for which I thank you for allowing me to provide stewardship. I close by again thanking the entire *JCMR* “village” for contributing to our success. Remember to also join us for our monthly *JCMR* Journal Club on the second Wednesday of the month at 11am ET!

Wishing you a happy, healthy, and safe 2023. We take great pride in the health care advances enabled by the ongoing advances in CMR. Remember to also take a deep breath every day to enjoy the moment.

## Data Availability

Data sharing not applicable to this article as no datasets were generated or analyzed.

## Abbreviations

APC: Article processing charge
CME: Continuing medical education
JCMR: Journal of Cardiovascular Magnetic Resonance
SCMR: Society for Cardiovascular Magnetic Resonance
